# Serum NPTX2 and cognitive impairment in geriatric diabetes

**DOI:** 10.17305/bb.2025.12632

**Published:** 2025-07-22

**Authors:** Kenan Çadırcı, Muharrem Bayrak, Alev Lazoğlu Özkaya, Gamze Nur Yılmaz, Nur Pak, Büşra Karahan, Hilal Kiziltunc Ozmen

**Affiliations:** 1 Department of Internal Medicine, Erzurum Faculty of Medicine, Health Sciences University, Erzurum, Türkiye; 2 Department of Biochemistry, Erzurum Faculty of Medicine, Health Sciences University, Erzurum, Türkiye; 3 Department of Child Health and Diseases Nursing, Institute of Health Sciences, Atatürk University, Erzurum, Türkiye; 4 Department of Internal Medicine Nursing, Institute of Health Sciences, Atatürk University, Erzurum, Türkiye; 5 Department of Radiation Oncology, Faculty of Medicine, Atatürk University, Erzurum, Türkiye

**Keywords:** Diabetes mellitus, geriatric patients, cognitive dysfunction, neuronal pentraxin 2, NPTX2.

## Abstract

Cognitive impairment is an increasingly common complication of diabetes, yet its underlying pathophysiological mechanisms remain poorly understood. Neuronal pentraxin 2 (NPTX2), a recently identified synaptic biomarker linked to cognitive disorders, has not previously been examined in relation to cognitive function in geriatric individuals with diabetes. This cross-sectional study enrolled 90 participants—46 geriatric patients with diabetes and 44 age-matched non-diabetic controls. Demographic and clinical data were collected for all participants. After informed consent, cognitive function was assessed with the Montreal Cognitive Assessment (MoCA) and the Mini-Mental State Examination (MMSE). Serum NPTX2 levels were measured by ELISA. No significant differences were found between the diabetic and control groups in age, sex, education level, marital status, smoking history, comorbid conditions, or polypharmacy. However, the groups differed significantly in MoCA scores (*P* < 0.001), MMSE scores (*P* ═ 0.028), and NPTX2 levels (*P* ═ 0.048); the diabetic group showed lower cognitive scores and biomarker levels. NPTX2 levels correlated positively with MoCA and MMSE scores and negatively with diabetes duration, patient age, and the presence of microvascular complications. In conclusion, cognitive function was significantly lower in geriatric patients with diabetes than in controls, and serum NPTX2 levels were significantly associated with cognitive performance. These findings suggest a possible role for NPTX2 in diabetes-related cognitive decline and support further investigation of its utility within a broader biomarker panel.

## Introduction

Diabetes mellitus is a serious metabolic disorder characterized by chronic hyperglycemia and is traditionally associated with well-known microvascular and macrovascular complications [1, 2]. However, advancements in diabetes management and increased life expectancy have led to a broader spectrum of diabetes-related comorbidities beyond these classical complications. Recent literature emphasizes the heightened risk and burden of additional conditions in individuals with diabetes, including an elevated incidence of specific cancers—particularly gastrointestinal and ovarian cancers—greater susceptibility to infections, liver disease, and various functional impairments. Notably, there is growing evidence of a strong association between diabetes and cognitive and affective disorders [3, 4]. Scientific studies increasingly report cognitive impairments in diabetic patients, encompassing deficits in memory, attention, concentration, verbal fluency, complex motor skills, processing speed, and executive functioning [5, 6].

Cognitive dysfunction, defined as a decline in one or more cognitive abilities, is a significant issue that negatively impacts quality of life by diminishing functional capacity across various domains. Early diagnosis and appropriate management of cognitive impairment may help slow its progression, thereby enhancing self-care abilities and overall quality of life in individuals with diabetes [7]. The relationship between diabetes and cognitive dysfunction is complex, with underlying mechanisms that remain to be fully elucidated. However, key pathophysiological factors are believed to include disruption of synaptic mitochondrial homeostasis in the brain, driven by pathological processes such as hyperglycemia, oxidative stress, inflammation, insulin resistance, and the accumulation of advanced glycation end products (AGEs). These factors contribute to synaptic dysfunction and neuronal loss, forming the core mechanisms linking diabetes to cognitive decline [8].

Synaptic mitochondria play a crucial role in meeting the high energy demands necessary for synaptic transmission, which is fundamental to learning and memory formation [9, 10]. These mitochondria are particularly vulnerable to AGE-induced damage, and their dysfunction is a significant contributor to age-related cognitive decline and synaptic degeneration [11]. Increasing evidence suggests that mitochondrial plasticity is critical to synaptic transmission, encompassing the synthesis and storage of neurotransmitters, synaptic vesicle trafficking, neurotransmitter release into the synaptic cleft, and subsequent recycling of neurotransmitters [12].

Neuronal pentraxins (NPTXs) are emerging as potential biomarkers for synaptic dysfunction [13]. This protein family includes NPTX1, NPTX2, and the neuronal pentraxin receptor (NPTXR), all of which are integral to neurotransmitter transmission, the regulation of synaptic function, synaptic plasticity, neuronal survival, and the modulation of excitatory synapses [14]. Notably, synaptic plasticity is essential for cognitive processes, including learning and memory [15]. Furthermore, evidence suggests that NPTXs are involved in intracellular processes such as mitochondrial dynamics, trafficking, and neuronal apoptosis [13].

NPTX2 is particularly important for the formation and stabilization of excitatory synapses that contain α-amino-3-hydroxy-5-methyl-4-isoxazolepropionic acid) (AMPA) glutamate receptors (GluRs). It regulates the quantity of AMPA receptors at synapses and facilitates their clustering, thereby playing a crucial role in synaptic plasticity, neuronal survival, and excitatory synaptic activity within the central nervous system [14]. Additionally, NPTX2 is significant in both developmental and adult synaptic plasticity [16]. These functions collectively enable the precise regulation of learning, memory formation and storage, information processing, and other fundamental neurological functions.

NPTX2 is a calcium (Ca^2+^)-dependent lectin that plays an important role in neurodevelopmental processes. This protein is also called neuronal activity-regulated pentraxin [17–19]. It is widely expressed in the brain, particularly in the hippocampus [17]. Parvalbumin (PV) interneurons are the most common interneuron subtype in the hippocampus. These γ-aminobutyric acid (GABA)–ergic interneurons are crucial for maintaining an optimal balance of excitation and inhibition [13] NPTX2 binds to the GluR ionotropic AMPA 4 (GluA4) subunit of AMPA receptors in PV interneurons. This binding helps maintain the excitatory–inhibitory balance. NPTX deficiency can disrupt this balance, leading to impairments in learning and memory. Given its central involvement in these processes, NPTX2 has garnered increasing attention in recent years for its potential role in neurodegenerative, neuropsychiatric, and other neurological disorders. Numerous studies have investigated its role in the pathophysiology of several other neurological diseases, such as schizophrenia [17], neuropathic pain [18], vascular dementia [19], Alzheimer’s disease [20], Parkinson’s disease [21], bipolar disorder [22], postoperative delirium [23, 24], epilepsy [25], amyotrophic lateral sclerosis, and frontotemporal dementia [26], as well as its effect on impaired cognitive function in combination with these diseases.

Glutamate is a vital excitatory neurotransmitter in the central nervous systems of mammals and humans. It facilitates neural activity through GluRs [27] and plays a significant role in regulating insulin secretion from pancreatic beta cells [28].

NPTX2, which operates through AMPA-type GluRs in the brain, plays a role in regulating insulin secretion from the beta cells of the islets of Langerhans. Research indicates that NPTX2 mRNA expression is reduced in the pancreatic islets of patients with type 1 diabetes [29]. One study demonstrated that the inhibition of LKB1, a regulator of 5’-adenosine monophosphate-activated protein kinase (AMPK) involved in cellular energy homeostasis, leads to an increase in NPTX2 expression and enhanced glutamatergic signaling, resulting from hyperplasia and an increase in pancreatic beta cell mass [30]. These findings suggest a complex, albeit not yet fully understood, pathophysiological relationship among NPTX2, AMPA-type GluRs, glutamate, neuronal plasticity, and insulin secretion from the pancreas. The unresolved aspects of this relationship may represent critical components of the pathophysiological processes linking diabetes to cognitive function decline associated with the condition.

To our knowledge, no studies have specifically examined the role of NPTX2 in the cognitive dysfunction associated with diabetes. While significant research has focused on cognitive impairment in diabetic patients, these studies have primarily utilized internationally validated cognitive assessment tools such as the MoCA and MMSE [31, 32]. To date, there has been no exploration of molecular biomarkers to assess cognitive decline or to elucidate the underlying mechanisms in this population. This study is significant as it represents the first effort to evaluate the association between serum NPTX2 levels and cognitive performance in individuals with diabetes. Given NPTX2’s established role in synaptic plasticity and its dysregulation in neurodegenerative diseases, it may also be implicated in the cognitive complications of diabetes. We hypothesize that lower serum NPTX2 levels are associated with impaired cognitive function in older adults with type 2 diabetes and may serve as a potential biomarker for diabetes-related cognitive decline. Therefore, the objective of this study is to investigate the relationship between serum NPTX2 concentrations and cognitive performance in elderly diabetic patients, with the aim of identifying early indicators of cognitive impairment and potential targets for intervention.

## Materials and methods

### Study design and population

This study utilized a single-center, descriptive, cross-sectional design conducted at the diabetes and general internal medicine outpatient clinics of a university hospital in Erzurum, Türkiye, from June 2024 to January 2025. Erzurum, the third-largest province in the Eastern Anatolia region, has an approximate population of 763,320. The hospital serves as a major referral center in the region, featuring 1042 beds, including 146 designated for geriatric patients. The research adhered to the principles outlined in the Declaration of Helsinki. All assessments were conducted through face-to-face interviews by a trained nurse to ensure consistency across evaluations and to obtain the most accurate and standardized data possible.

### Study population

Men and women aged 65 and older who attended routine check-ups and were either diagnosed with diabetes or identified as nondiabetic controls were included in this study. The inclusion criteria required participants to provide voluntary informed consent, possess the ability to read and write in Turkish, have completed at least five years of formal education, and have no hearing or speech impairments. Participants had to be 65 years of age or older.

Exclusion criteria consisted of a known diagnosis of any psychiatric or neurological disorder, current use of psychiatric medications, the presence of kidney or liver disease, inability to communicate or follow instructions, a history of brain surgery, evidence of focal brain lesions on neuroimaging, a history of head trauma, or current alcohol or substance dependence. Exclusion decisions were based on a comprehensive review of participants’ medical records and brief clinical interviews conducted at enrollment. This methodology aimed to minimize the confounding effects of preexisting neuropsychiatric conditions, thereby enabling a focused evaluation of the relationship between diabetes and cognitive function in the context of serum NPTX2 levels.

### Demographics and clinical information of the participants

The medical records of all participants were meticulously reviewed to gather data on age, sex, educational attainment, marital status, comorbidities, current medications, duration of illness, smoking history, body mass index (BMI), and blood pressure.

### Neurological assessment

#### Application of neurocognitive tests

##### The Montreal Cognitive Assessment (MoCA)

The MoCA, developed by Nasreddine et al. [33], is a succinct yet comprehensive cognitive screening tool recognized for its high sensitivity and specificity in identifying early-stage cognitive impairment. It assesses eight cognitive domains: attention and concentration, executive function, memory, language, visuoconstructional skills, conceptual thinking, calculations, and orientation. The total possible score ranges from 0 to 30, with higher scores indicating superior cognitive performance.

MoCA test outcomes may vary due to factors such as age, cultural background, education level, and the tool’s adaptation, validity, and reliability. Research has been conducted on the application of MoCA in Türkiye [34]. The original MoCA recommends a cutoff score of 26 to denote normal cognition; however, the Turkish validation study by Selekler et al. [34] determined a threshold of 21. In this study, scores of 21 or higher were classified as normal, while scores below 21 indicated cognitive decline within the Turkish population.

##### The Mini Mental State Examination (MMSE) test

Originally developed by Folstein et al. in 1975, [35], the Mini-Mental State Examination (MMSE) is a widely utilized screening tool for the rapid and practical assessment of cognitive function. The examination consists of 11 questions that assess five core cognitive domains: orientation, registration, attention and calculation, recall, and language. Similar to the MoCA, MMSE scores range from 0 to 30, with higher scores indicating better cognitive performance. A validity and reliability study conducted by Güngen et al. [36] specifically examined the MMSE for the Turkish population, focusing on its effectiveness in detecting mild dementia among individuals with formal education. According to their findings, a score of 24 or above is considered indicative of normal cognitive function in the Turkish population, whereas scores below 24 suggest cognitive impairment.

### Measurements of serum NPTX2

Fasting blood samples were collected between 08:00 and 10:00 a.m., after a period of rest with participants seated. Venipuncture was performed by trained personnel using vacutainer tubes, with blood drawn from the antecubital vein. For serum NPTX2 level measurement, samples were placed in yellow-capped biochemistry tubes and allowed to clot at room temperature. Subsequently, the samples were centrifuged at 3000 rpm for 10 min to separate the serum, which was stored at –80 ^∘^C until analysis.

Prior to analysis, serum samples were thawed under controlled conditions, and all assays were conducted in a single batch at the medical biochemistry laboratory of the same university hospital. NPTX2 concentrations were determined using a BT (China) ELISA kit (Human NPTX2, Catalog No. E4709Hu) and a Rel Assay automated ELISA reader (BiobaseBiodusty, Shandong, China), following the manufacturer’s instructions. The intra-assay and inter-assay coefficients of variation (CVs) were reported as <8% and <10%, respectively. The kit’s detection range for NPTX2 was 0.05–20 ng/mL. ELISA was selected as it is a validated gold standard method for the quantitative detection of low-abundance proteins in serum, blood, plasma, and other body fluids. To minimize variability and nonspecific effects, all samples were analyzed in duplicate under identical conditions within a single assay batch.

A box plot evaluation indicated that only three values met the criteria for mild outliers (data not provided). However, these values were retained as they fell within the physiologically plausible range and did not violate the assumptions of normality. Three mild outliers (8.74, 9.21, and 12.67 ng/mL) in serum NPTX2 levels were identified based on the 1.5 × IQR criterion; these values were retained in the final analysis as they were within a biologically plausible range. Two of these participants were in the diabetic group, and one was non-diabetic.

### Ethical statement

The study received approval from the Scientific Research Ethics Committee of Health Sciences University, Erzurum Medical Faculty (Decision No. BAEK-12-234). The research objectives were thoroughly communicated to all participants, and written informed consent was obtained from each individual before their inclusion in the study.

### Statistical analysis

Statistical analyses were conducted using the Statistical Package for the Social Sciences (SPSS) for Windows, version 22.0 (SPSS Inc., Chicago, IL, USA). The effect size was calculated at a large level (Cohen’s *d* ═ 0.8) using the G-Power 3.1.9.7 program for *t*-tests, given the absence of a reference article. With an alpha level (*α*) set at 0.05 and a power (1-*β*) of 0.90, the minimum sample size required was determined to be 68 participants, with 34 in both the control and experimental groups [37]. Descriptive statistics, including means, standard deviations, and percentages, were employed to summarize the data. The Kolmogorov–Smirnov test assessed the normality of numerical variables. The Mann–Whitney *U* test was applied for comparisons between two independent groups with non-normally distributed data. Differences in categorical variables between groups were analyzed using the chi-square test. Spearman’s correlation coefficients (*r*) were calculated to evaluate the relationships between numerical variables. All results were interpreted within a 95% confidence interval, with statistical significance defined as *P* < 0.05.

## Results

### Participants’ baseline clinical characteristics

The sociodemographic and clinical characteristics of the study population are presented in Tables 1 and 2. A total of 90 individuals participated in the research, comprising 46 individuals with type 2 diabetes and 44 controls. The diabetic group included 22 men (47.8%) and 24 women (52.2%), while the non-diabetic group consisted of 22 men (50%) and 22 women (50%). The median age of the diabetic group was 67.5 years (interquartile range [IQR]: 65–75), with a median BMI of 29.9 kg/m^2^ (IQR: 26.6–33.9). In contrast, the control group had a median age of 68.5 years (IQR: 65–75) and a median BMI of 26.5 kg/m^2^ (IQR: 23.5–28.5). The diabetic group exhibited a significantly higher BMI (*P* < 0.001), while no significant differences were observed between the groups regarding age (*P* ═ 0.650) or sex (*P* ═ 0.502). The increased BMI in diabetic patients compared to non-diabetic individuals may be attributed to insulin resistance, increased visceral fat accumulation, the weight-gain effects of certain antidiabetic medications, and lifestyle factors such as decreased physical activity. Regarding educational attainment, 67.4% of the diabetic group had completed at least primary education (a minimum of five years), 28.3% had completed middle school, and 4.3% had attained university level or higher education. The comparable values in the control group were 65.9%, 15.9%, and 18.2%, respectively, with no significant differences detected between the groups. Regarding marital status, 74% of participants in the diabetic group were married, while 26% were widowed. In comparison, 65.9% of the control group were married and 34.1% were widowed; these differences were not statistically significant. With respect to comorbid conditions, the prevalence of hypertension was 76% in the diabetic group and 77% in the control group (*P* ═ 0.547). The corresponding prevalences for other conditions were as follows: chronic obstructive pulmonary disease (COPD) at 30.5% and 34% (*P* ═ 0.442); coronary artery disease (CAD) at 58.6% and 54.5% (*P* ═ 0.253); congestive heart failure (CHF) at 34.7% and 29.5% (*P* ═ 0.380); and atrial fibrillation (AF) at 30.4% and 25% (*P* ═ 0.367). None of these differences were statistically significant. Polypharmacy was observed in 56.5% of the diabetic group and 50% of the control group, with this difference also being statistically insignificant. In summary, there were no significant differences between the two groups regarding age, sex, educational level, marital status, smoking history, comorbid diseases, or polypharmacy (see Tables 1 and 2). The diabetic group exhibited significantly higher systolic and diastolic blood pressure values compared to the non-diabetic group (SBP: *P* < 0.001; DBP: *P* ═ 0.016) (Table 2). The median (IQR) MoCA score was significantly higher in the control group (21 [16.2–23]) than in the diabetic group (12 [8–15]) (*P* < 0.001). Similarly, the median (IQR) MMSE score was significantly greater in the control group (21 [18–23]) compared to the diabetic group (18 [13–23]) (*P* ═ 0.028) (Table 2). The significantly higher MoCA and MMSE scores in non-diabetic individuals may be attributed to the absence of chronic hyperglycemia-related neurodegeneration, vascular complications, and insulin resistance, all of which are known to contribute to cognitive decline in patients with diabetes.

**Table 1:** Comparison of the sociodemographic characteristics of the non-diabetic patients and diabetic patients

**Variables**	**Group**			
	**Non-diabetic patients (*n* ═ 44)**	**Diabetic patients (*n* ═ 46)**	**Overall (*n* ═ 90)**	***P* value**
*Demographic characteristics*				
Age (year), median (IQR)	68.5 (65–75)	67.5 (65–75)	68 (65–75)	0.650
*Sex*				
Men, *n*(%)	22 (50)	22 (47.8)	44 (100)	0.502
Women, *n*(%)	22 (50)	24 (52.2)	46 (100)	
BMI (kg/m^2^), median (IQR)	26.5 (23.5–28.5)	29.9 (26.6–33.9)	27.75 (24.65–31.13)	<0.001
*Educational level*				
Primary school or above (at least >5 year), *n*(%)	29 (65.9)	31 (67.4)	60 (66.7)	0.292
Middle school, *n*(%)	7 (15.9)	13 (28.3)	20 (22.2)	
High school or above, *n*(%)	8 (18.2)	2 (4.3)	10 (11.1)	
*Marital status*				
Married, *n*(%)	29 (65.9)	34 (74)	63 (70)	0.275
Separated or divorced, *n*(%)	0	0	0	
Widowed, *n*(%)	15 (34.1)	12 (26)	27 (30)	
Never married, *n*(%)	0	0	0	
*Smoking history*				
Smoker, *n*(%)	14 (31.8)	15 (32.6)	29 (32.2)	0.970
Nonsmoker, *n*(%)	23 (52.3)	24 (52.2)	47 (52.2)	
Ex-smoker, *n*(%)	7 (15.9)	7 (15.2)	14 (15.6)	

‘Overall’ refers to pooled data from both non-diabetic and diabetic patients. BMI: Body mass index.

**Table 2:** Comparison of the clinical characteristics of the non-diabetic and diabetic patients

**Variables**	**Group**			
	**Non-diabetic patients** **(*n* ═ 44)**	**Diabetic patients** **(*n* ═ 46)**	**Overall** **(*n* ═ 90)**	***P* value**
*Type of comorbid disease*				
Hypertension, *n*(%)	34 (77)	35 (76)	69 (76.6)	0.547
COPD, *n*(%)	15 (34)	14 (30.5)	29 (32.2)	0.442
CAD, *n*(%)	24 (54.5)	27 (58.6)	51 (56.6)	0.253
CHF, *n*(%)	13 (29.5)	16 (34.7)	29 (32.2)	0.380
AF, *n*(%)	11 (25)	14 (30.4)	25 (27.7)	0.367
Polypharmacy, *n*(%)	22 (50)	26 (56.5)	48 (53.3)	0.342
Peripheral artery disease	4 (9.1)	1 (2.1)	5 (5.6)	0.167
*Tension arterial*				
Systolic blood pressure (mm Hg) median (IQR)	128 (124–132)	131.5 (128–134)	129 (127–132)	**<0.001**
Diastolic blood pressure (mm Hg) median (IQR)	86.5 (83–89)	87 (84–92)	87 (84–91)	**0.016**
MoCA median (IQR)	21 (16.2–23)	12 (8–15)	15 (10.75–21)	**<0.001**
MMSE median (IQR)	21 (18–23)	18 (13–23)	19.5 (15.75–23)	**0.028**

‘Overall’ refers to pooled data from both non-diabetic and diabetic patients. COPD: Chronic obstructive pulmonary disease; CAD: Coronary artery disease; CHF: Congestive heart failure; AF: Atrial fibrillation; MoCA: Montreal cognitive assessment; MMSE: Mini-mental state examination.

### Diabetes-related characteristics of the patients

The diabetic group exhibited significantly higher systolic and diastolic blood pressure values compared to non-diabetic individuals (Table 2). This finding aligns with the established association between type 2 diabetes and increased cardiovascular risk profiles.

We evaluated various diabetes-related characteristics, including the duration of diabetes, treatment modalities, and accompanying microvascular complications. Patients were categorized into four groups based on the duration of diabetes: 0–5 years (3 patients), 6–10 years (15 patients), 11–15 years (22 patients), and more than 16 years (6 patients). Regarding treatment, patients were classified into four groups: lifestyle modification (3 patients), oral hypoglycemic agents only (8 patients), insulin only (20 patients), and a combination of insulin and oral hypoglycemic agents (15 patients). An assessment of microvascular complications revealed that 21 patients had nephropathy, 20 had retinopathy, and 41 had neuropathy. The diabetes-related characteristics of the patients are summarized in Table 3.

**Table 3:** Diabetes-related characteristics of diabetic patients

**Variables**	**Diabetic patients** **(*n* ═ 46)**
***Duration of diabetes***	
0–5 year, *n*(%)	3 (6.5)
6–10 year, *n*(%)	15 (32.6)
11–15 year, *n*(%)	22 (47.9)
>16 year, *n*(%)	6 (13)
*Treatment of diabetes*	
Lifestyle modification, *n*(%)	3 (6.5)
Oral medications only, *n*(%)	8 (17.4)
Insulin only, *n*(%)	20 (43.5)
Oral medications+ insulin, *n*(%)	15 (32.6)
*Microvascular complications*	
Nephropathy, *n*(%)	21 (45.6)
Retinopathy, *n*(%)	20 (43.4)
Neuropathy, *n*(%)	41 (89.1)

There were no significant differences among the groups regarding TSH (*P* ═ 0.142), vitamin B12 (*P* ═ 0.688), eGFR (*P* ═ 0.306), or creatinine (*P* ═ 0.704) levels. However, significant differences were observed in fasting plasma glucose (*P* < 0.001), blood hemoglobin A1c (HbA1c) (*P* < 0.001), high-density lipoprotein cholesterol (*P* ═ 0.014), and triglyceride levels (*P* < 0.001). Additionally, NPTX2 levels differed significantly between the groups (*P* ═ 0.048). The laboratory findings for the study groups are presented in Table 4.

**Table 4:** Laboratory findings of the study groups [median, (IQR)]

**Variables**	**Groups**		**Overall (*n* ═ 90)**	***P* value**
	**Diabetic patients**	**Non-diabetic patients**		
	**PR (95% CI)**	**PR (95% CI)**	**PR (95% CI)**	
NPTX2 (ng/mL)	1.15 (0.81–1.58)	1.41 (1.03–2.45)	1.29 (0.88–1.82)	**0.048**
Fasting plasma glucose (mg/dL)	176.5 (124–242.2)	84.5 (78–100.5)	112 (84–180)	**<0.001**
Blood hemoglobin A1c(%)	9.2 (7.5–11)	5.7 (5.5–5.9)	6.35 (5.67–9.25)	**<0.001**
Blood creatinine (mg/dL)	0.92 (0.76–1.19)	0.92 (0.74–1.26)	0.92 (0.75–1.22)	0.704
eGFR (mL/min/1,73 m^2^)	82.5 (54–101.6)	81.8 (50.3–88.5)	82.2 (53.2–96.2)	0.306
Sodium (mmol/L)	139 (136–141)	141 (139–142)	139 (137–142)	0.148
Potassium (mmol/L)	4.3 (3.6–4.6)	4.2 (3.7–4.4)	4.3 (3.6–4.6)	0.455
Calcium (mg/dL)	9.25 (8.8–9.8)	9.4 (8.7–9.7)	9.4 (8.8–9.8)	0.954
Albumin (g/L)	40.5 (35.7–42)	39 (35.2–42)	39.5 (35.75–42.0)	0.379
Low density lipoprotein cholesterol (mg/dL)	120 (89–145)	110.5 (87.2–134.2)	117.5 (88–140.2)	0.696
High density lipoprotein cholesterol (mg/dL)	37.8 (31.3–44.6)	43.5 (35.3–52.1)	41.3 (33.6–50.8)	**0.014**
Triglycerides (mg/dL)	150 (104.5–241.5)	94.5 (81.7–133)	121 (87–175)	**<0.001**
Total cholesterol (mg/dL)	180 (145.7–217.7)	176.5 (152.2–220.2)	179 (147.5–218.7)	0.886
TSH (mIU/L)	1.23 (0.87–1.82)	1.16 (0.61–1.9)	1.20 (0.7–1.8)	0.142
Vitamin B12 (pg/mL)	339.5 (269.7–398.7)	315.5 (252.2–376.7)	326 (259–389)	0.688
Ferritin (ng/mL)	86.4 (27.6–156.4)	62.3 (23.6–98.2)	77.6 (24.9–122)	0.098
25-hidroksi vitamin D (ng/mL)	17.3 (12.6–30.8)	13.6 (11.2–25.1)	15.4 (11.6–28.1)	0.169

‘Overall’ refers to pooled data from both non-diabetic and diabetic patients. NPTX2: Neuronal pentraxin 2; eGFR: Estimated glomerular filtration rate; TSH: Thyroid-stimulating hormone; PR: Percentile range 95% confidence intervals (CI) are shown.

NPTX2 levels exhibited a positive correlation with MMSE scores (*r* ═ 0.235, *P* ═ 0.026) and MoCA scores (*r* ═ 0.242, *P* ═ 0.022). Conversely, NPTX2 levels were negatively correlated with the duration of diabetes (*r* ═ −0.219, *P* ═ 0.038), patient age (*r* ═ −0.289, *P* ═ 0.006), nephropathy (*r* ═ −0.253, *P* ═ 0.016), retinopathy (*r* ═ −0.281, *P* ═ 0.007), polypharmacy (*r* ═ −0.279, *P* ═ 0.008), CHF (*r* ═ −0.216, *P* ═ 0.041), and AF (*r* ═ −0.283, *P* ═ 0.007). The correlations of NPTX2 with the numerical variables are summarized in Table 5.

**Table 5:** Correlation of the NPTX2 with numerical variables

**Variables**	***r***	***P***
MMSE	0.235	0.026
MoCA	0.242	0.022
Duration of diabetes (year)	−0.219	0.038
Age (year)	−0.289	0.006
Nephropathy	−0.253	0.016
Retinopathy	−0.281	0.007
Polypharmacy	−0.279	0.008
CHF	−0.216	0.041
AF	−0.283	0.007

MMSE: Mini-mental state examination; MoCA: Montreal cognitive assessment; CHF: Chronic heart failure; AF: Atrial fibrillation.

Although the correlation coefficient between NPTX2 and MoCA was relatively modest (*r* ≈ 0.24), it achieved statistical significance, indicating that this relationship is unlikely to be coincidental. This finding may reflect the intricate, multifactorial nature of cognitive decline related to diabetes, which results from a combination of vascular, metabolic, and neuroinflammatory processes. In this context, even biomarkers with modest effect sizes can yield clinically relevant insights, especially when they represent specific disease aspects, such as synaptic dysfunction. It is also noteworthy that small but statistically significant correlations are commonly observed in early biomarker research and may retain diagnostic or prognostic significance when integrated with other clinical variables.

MoCA scores demonstrated a positive correlation with MMSE scores (*r* ═ 0.502, *P* < 0.001), NPTX2 levels (*r* ═ 0.242, *P* ═ 0.022), and educational attainment (*r* ═ 0.250, *P* ═ 0.017). Conversely, MoCA scores exhibited negative correlations with the duration of diabetes (*r* ═ −0.546, *P* < 0.001), patient age (*r* ═ −0.222, *P* ═ 0.035), BMI (*r* ═ −0.279, *P* ═ 0.008), fasting plasma glucose (*r* ═ −0.436, *P* < 0.001), HbA1c (*r* ═ −0.471, *P* < 0.001), triglycerides (*r* ═ −0.303, *P* ═ 0.004), nephropathy (*r* ═ −0.335, *P* < 0.001), retinopathy (*r* ═ −0.368, *P* < 0.001), and neuropathy (*r* ═ −0.406, *P* < 0.001). The correlations between MoCA scores and these numerical variables are summarized in Table 6.

**Table 6:** Correlation of the MoCA score with numerical variables

**Variables**	***r***	***P***
MMSE	0.502	<0.001
NPTX2 (ng/mL)	0.242	0.022
Duration of diabetes (year)	−0.546	<0.001
Education level	0.250	0.017
Age (year)	−0.222	0.035
BMI (kg/m^2^)	−0.279	0.008
Fasting plasma glucose (mg/dL)	−0.436	<0.001
Blood hemoglobin A1c(%)	−0.471	<0.001
Triglycerides (mg/dL)	−0.303	0.004
Nephropathy	−0.335	<0.001
Retinopathy	−0.368	<0.001
Neuropathy	−0.406	<0.001

MMSE: Mini-mental state examination; NPTX2: Neuronal pentraxin 2; BMI: Body mass index.

MMSE scores were positively correlated with MoCA scores (*r* ═ 0.502, *P* < 0.001), NPTX2 levels (*r* ═ 0.235, *P* ═ 0.026), educational attainment (*r* ═ 0.260, *P* ═ 0.013), and vitamin B12 levels (*r* ═ 0.214, *P* ═ 0.004). In contrast, MMSE scores were negatively correlated with the duration of diabetes (*r* ═ −0.217, *P* ═ 0.040), patient age (*r* ═ −0.214, *P* ═ 0.041), fasting plasma glucose (*r* ═ −0.235, *P* ═ 0.026), HbA1c (*r* ═ −0.224, *P* ═ 0.034), and polypharmacy (*r* ═ −0.258, *P* ═ 0.014). The correlations between MMSE scores and the numerical variables are presented in Table 7.

**Table 7:** Correlation of the MMSE score with numerical variables

**Variables**	***r***	***P***
MoCA	0.502	<0.001
NPTX2	0.235	0.026
Education level	0.260	0.013
Vitamin B12 (pg/mL)	0.214	0.004
Duration of diabetes (year)	−0.217	0.040
Age (year)	−0.214	0.041
Fasting plasma glucose (mg/dL)	−0.235	0.026
Blood hemoglobin A1c(%)	−0.224	0.034
Polypharmacy	−0.258	0.014

MoCA: Montreal cognitive assessment; NPTX2: Neuronal pentraxin 2.

## Discussion

The global prevalence of diabetes is rapidly increasing. Nevertheless, advancements in therapeutic options, earlier detection of potential micro- and macrovascular complications, and more effective treatments have contributed to increased life expectancy among patients. However, this extended life expectancy has introduced new clinical challenges, particularly self-care difficulties and functional and cognitive impairments. Cognitive impairment adversely affects quality of life by diminishing functional capacity, which significantly impacts diabetic patients. These individuals must adapt to lifestyle changes and consistently utilize oral or injectable antidiabetic therapies for optimal management, which can lead to severe complications such as hypoglycemia, treatment failure, and hospitalization. Current approaches, therefore, involve elucidating the pathophysiology of diabetes-related cognitive failure and identifying the molecular bases that might play a key role in that relationship. They also involve identifying drugs or approaches capable of positively impacting treatment in light of those molecular bases, determining accurate, early and consistent diagnostic methods and biomarkers, and establishing optimal approaches to complications that may arise [8, 38, 39].

According to the National Diabetes Statistics Report (2024), the prevalence of diabetes among adults increases with age, currently affecting 29.2% of individuals aged 65 and older [40]. The American Diabetes Association advises that individuals with diabetes aged 65 and above should be screened for cognitive function impairment during their initial visit and annually thereafter [41]. Consequently, this study includes diabetic patients over 65 years of age, as potential cognitive decline may exacerbate adverse outcomes related to inadequate self-care and treatment.

This study included two groups, a diabetic group and a control group, both aged over 65 years, with no statistically significant differences in terms of age, sex, education level, marital status, smoking history, accompanying additional diseases, or polypharmacy use. Significant cognitive function loss, as determined via the MoCA and MMSE scores, was observed in the diabetic group. The literature highlights that diabetes is a substantial risk factor for cognitive function disorders [42, 43]. Adults with type 2 diabetes mellitus have reported a 1.46- to 2.48-fold increased risk of developing dementia compared to the general population [44]. Consistent with these findings, our study documented a considerable decline in cognitive function among geriatric patients with diabetes.

In addition to being used to diagnose and treat patients with diabetes [45–48], prediabetes [49, 50], insulin resistance [51, 52], and metabolic syndrome [53], which represent different stages of glucose metabolism disorders, are associated with pathological changes that might take place in regions of the brain associated with cognitive functions and poorer cognitive performance. Evidence also suggests that early-onset type 1 diabetes [54], hypoglycemic episodes during treatment [55], and glycemic fluctuations [56] correlate with reduced cognitive performance. We acknowledge that the exclusion of hypoglycemic episodes and glycemic fluctuations from our evaluation constitutes a limitation of this study.

Thyroid hormones and vitamin B12 are essential for brain development and function, influencing processes such as neuron myelination and neurotransmitter synthesis [57, 58]. Given the significant correlation between vitamin B12 and thyroid hormone levels, neuropsychiatric symptoms, and dementia-related conditions [49, 59], as well as the association between elevated creatinine levels and impaired cognitive function [60], this study ensured that TSH, vitamin B12, and creatinine levels remained within normal ranges. No significant differences were observed between the study groups concerning these parameters.

The diagnostic evaluation of cognitive function in individuals with diabetes parallels the approach used for nondiabetic individuals. The American Diabetes Association guidelines recommend cognitive screening tools such as the MoCA and MMSE when dementia is suspected [61]. Both assessments are internationally recognized and validated; however, their effectiveness may be compromised by the requirement for administration by trained professionals and the literacy levels of patients. In this study, measures were taken to enhance the reliability and standardization of cognitive assessments. Specifically, only participants with a minimum of five years of formal education were included, and all tests were administered by trained professionals to ensure consistency and accuracy in the results.

An increasing body of research has identified NPTX2 as a prognostic marker for cognitive decline associated with neurodegenerative, neuropsychiatric, and other neurological disorders [17, 18, 22, 25]. Consistent with these findings, the present study revealed a significant difference in NPTX2 levels between the diabetic and control groups. Additionally, NPTX2 levels were positively correlated with both MMSE and MoCA scores. Although these associations were statistically significant, the correlation coefficients were modest, indicating that NPTX2 may represent only a partial contribution to the complex mechanisms underlying cognitive performance. Notably, NPTX2 and insulin share similar roles in maintaining normal synaptic structure and function, both positively influencing neuronal survival, synaptic plasticity, and cognitive processes such as learning and memory [62]. Based on these observations, we propose that the cognitive decline observed in the diabetic group, alongside reduced NPTX2 levels, may be partially attributed to decreased insulin levels and/or impaired insulin function commonly seen in diabetes patients.

Patients with peripheral microvascular complications—specifically retinopathy and nephropathy—exhibit a heightened risk of cognitive decline compared to those without such complications [63]. This study conducted a correlation analysis to investigate the relationships between diabetic microvascular complications and cognitive function. The findings revealed negative correlations between MoCA scores and the presence of nephropathy, retinopathy, and neuropathy, corroborating results from previous research. A study by Öktem et al. found that MoCA scores were significantly lower in individuals with diabetic retinopathy compared to healthy controls. Additionally, this study identified a notably reduced gray matter volume in the right insula among the retinopathy group relative to the control group [64].

In this study, we found that MoCA scores were negatively correlated with the duration of diabetes, fasting plasma glucose levels, and HbA1c levels. These results suggest that cognitive function deteriorates with longer disease duration, increasing patient age, and inadequate glycemic control. Consistent with prior research [65], our findings highlight the progressive and adverse effects of poorly managed, long-term diabetes on cognitive performance.

Directly comparing our findings with existing literature is challenging, as this study is, to the best of our knowledge, the first to evaluate cognitive function using a biomolecular marker instead of relying solely on standardized assessments such as the MoCA and MMSE.

Several limitations must be acknowledged in this study. First, the analysis did not consider the potential impact of hypoglycemic episodes and glycemic fluctuations, which may affect cognitive outcomes. Second, the cognitive effects of various antidiabetic medications were not evaluated individually. Third, while the exclusion of participants with preexisting neurological or psychiatric conditions aimed to reduce confounding variables, it may also restrict the applicability of the findings to the broader diabetic population, where such comorbidities are prevalent. Future research should address these factors to offer a more comprehensive understanding of the interactions among diabetes, cognitive function, and biomarkers like NPTX2. Additionally, NPTX2 is expressed not only in the central nervous system but also in pancreatic islet cells. Given the potential influence of metabolic disorders such as diabetes, serum NPTX2 levels may reflect both central and peripheral sources. This consideration is essential for the interpretation of our results.

## Conclusion

This study is the first to evaluate the association between serum NPTX2 levels and cognitive performance in individuals with diabetes. The results of the MoCA and MMSE tests in this study demonstrated a decline in cognitive function among geriatric diabetic patients. Additionally, NPTX2 levels differed significantly between the diabetic and control groups. The relationship observed between NPTX2 levels and cognitive impairment in diabetic patients provides preliminary evidence for a possible role of this biomarker in the pathophysiology of diabetes-related cognitive decline. Further in-depth research into the molecular pathways through which NPTX2 may influence cognitive function is needed to determine its clinical significance and potential utility in diagnosis or monitoring. A clearer understanding of the role of NPTX2 could also support its use as a biomarker for diagnosis and progression prediction, complementing internationally recognized cognitive assessment tools. Therefore, when the pathophysiological role of NPTX2 levels in cognitive impairment in patients with diabetes is fully proven, the current biomarkers may guide both rapid and early diagnosis and the development of an effective treatment modality via NTPX2.

## Data Availability

The datasets used in the study are available from the corresponding author upon reasonable request.
